# Relationship of cortisol levels and genetic polymorphisms to antidepressant response to placebo and fluoxetine in patients with major depressive disorder: a prospective study

**DOI:** 10.1186/s12888-014-0220-0

**Published:** 2014-08-03

**Authors:** Raúl Ventura-Juncá, Adriana Symon, Pamela López, Jenny L Fiedler, Graciela Rojas, Cristóbal Heskia, Pamela Lara, Felipe Marín, Viviana Guajardo, A Verónica Araya, Jaime Sasso, Luisa Herrera

**Affiliations:** Programa de Genética Humana, Instituto de Ciencias Biomédicas, Facultad de Medicina, Universidad de Chile, Independencia 1027, Independencia, Santiago Chile; Escuela de Psicología, Universidad de Los Andes, San Carlos de Apoquindo 2200, Las Condes, Santiago, Chile; Clínica Psiquiátrica Universitaria, Hospital Clínico Universidad de Chile, Av. La Paz 1003, Recoleta, Santiago, Chile; Laboratorio de Neuroplasticidad y Neurogenética, Departamento de Bioquímica y Biología Molecular, Universidad de Chile, Calle Sergio Livingstone Pohlhammer 1007 (ex Olivos), Independencia, Santiago, Chile; Departamento de Endocrinología, Universidad de Chile, Santos Dumont 999, Independencia, Santiago, Chile; Instituto de Investigaciones Farmacológicas y Toxicológicas (IFT), Facultad de Medicina, Universidad de Chile, Santiago, Chile

**Keywords:** Major depressive disorder, Salivary cortisol, Placebo, Fluoxetine, Antidepressants, Polymorphisms

## Abstract

**Background:**

Increased cortisol levels and genetic polymorphisms have been related to both major depressive disorder and antidepressant treatment outcome. The aim of this study is to evaluate the relationship between circadian salivary cortisol levels, cortisol suppression by dexamethasone and genetic polymorphisms in some HPA axis-related genes to the response to placebo and fluoxetine in depressed patients.

**Methods:**

The diagnosis and severity of depression were performed using the Mini International Neuropsychiatric Interview (M.I.N.I.) and Hamilton depression scale (HAM-D_17_), respectively. Euthyroid patients were treated with placebo (one week) followed by fluoxetine (20 mg) (two months). Severity of depression was re-evaluated after placebo, three weeks and two months of fluoxetine treatments. Placebo response was defined as HAM-D_17_ score reductions of at least 25% and to < 15. Early response and response were reductions of at least 50% after three weeks and two months, and remission with ≤ 7 after two months. Plasma TSH, free-T4, circadian salivary cortisol levels and cortisol suppression by dexamethasone were evaluated. Seven genetic polymorphisms located in the Corticotrophin-releasing-hormone-receptor-1 (rs242939, rs242941, rs1876828), Corticotrophin-releasing-hormone-receptor-2 (rs2270007), Glucocorticoid-receptor (rs41423247), FK506-binding-protein-5 (rs1360780), and Arginine-vasopressin (rs3729965) genes were determined. Association analyses between response to placebo/fluoxetine and polymorphism were performed by chi-square or Fisher exact test. Cortisol levels were compared by *t*-test, ANOVA and the general linear model for repeated measures.

**Results:**

208 depressed patients were recruited, 187 of whom were euthyroid. Placebo responders, fluoxetine responders and remitters exhibited significantly lower circadian cortisol levels than those who did not respond (p-values of 0.014, 0.008 and 0.021 respectively). Patients who abandoned treatment before the third week also exhibited a trend to low cortisol levels (p = 0.057). The polymorphisms rs242939 (CRHR1) and rs2270007 (CRHR2) were not in Hardy-Weinberg equilibrium. Only the rs242939 polymorphism (CRHR1) exhibited association with early response (three weeks) to fluoxetine (p-value = 0.043). No other association between outcomes and polymorphisms was observed.

**Conclusions:**

These results support the clinical relevance of low salivary cortisol levels as a predictor of antidepressant response, either to placebo or to fluoxetine. Only one polymorphism in the CRHR1 gene was associated with the early response. Other factors may be involved in antidepressant response, although further studies are needed to identify them.

**Electronic supplementary material:**

The online version of this article (doi:10.1186/s12888-014-0220-0) contains supplementary material, which is available to authorized users.

## Background

Major depressive disorder (MDD) [MIM 608516] is a highly prevalent mental disorder, characterized by depressed mood and loss of interest or pleasure in daily activities, often accompanied by high suicide rates. Lifetime prevalence of MDD in Chile is high, with an average of approximately 10% [1]. The recurrence risk of depression is also high and correlates with the number of previous episodes. Consequently, to avoid recurrence once depression has been diagnosed, it must be promptly and efficiently treated [2].

Nearly 40% of patients do not respond to the antidepressants recommended as a first line of treatment [3] and approximately 40% accomplish full remission [4]. Antidepressant therapeutic action is typically observed after 2–4 weeks of treatment. Therefore when an antidepressant treatment fails, there is a long delay until insufficient outcome can be assumed with some confidence, and as a result many patients lose time with ineffective antidepressant therapies. For these reasons, it is essential to find reliable markers that can help to predict the antidepressant outcome and to design effective personalized therapies.

The most common antidepressants currently available, including fluoxetine (FLX), are targeted to the monoaminergic systems [5,6]. The effects of antidepressants on monoamines are observed within a few hours. Intriguingly, the therapeutic response is observed with a delay of several weeks after the initiation of treatment [7]. This indicates that something beyond normalization of monoamines is required to accomplish the clinical antidepressant response. Hyperactivity of the HPA axis has been observed in patients with major depressive disorder and with poorer antidepressant outcome [8,9]. Moreover, elevated cortisol levels and no suppression of cortisol secretion after dexamethasone suppression test (DST) have been associated with worse antidepressant treatment outcome, relating the HPA axis dysregulation to the response [10]. This suggests that patients resistant to antidepressant treatment may represent a biologically distinct group [8,9].

On the other hand, 30-40% of patients with MDD respond to placebo [11]. Although the mechanisms underlying the placebo effects are still unknown, neurobiological changes such as neuroimaging differences between placebo responders and non-responders have been observed [12,13]. Recently low cortisol levels were reported in depressed patients with early life stress experiences that responded to placebo treatment, suggesting that the mechanisms of placebo effect could involve HPA axis activity [14]. Hence responsiveness to either placebo or antidepressant treatments may be related to the extent of the dysregulation of the HPA axis and to the ability to normalize the hypothalamic-pituitary-adrenal (HPA) axis function [14].

In addition, responsiveness could be influenced by other factors such as genetic polymorphisms or epigenetic modifications that interfere with the normal function of some genes of the HPA axis, limiting the normalization induced by antidepressants. For instance, polymorphisms in corticotrophin releasing hormone receptor 1 and 2 (CRHR1 and CRHR2), glucocorticoid receptor (GR), FK506-binding protein 5 (FKBP5), and arginine vasopressin (AVP) have been previously associated with HPA (dys) regulation, MDD and/or antidepressant response [15-19]. Briefly, in the GR gene some of the SNPs have been associated with depression and antidepressant effects. One of them, the BclI polymorphism, has been associated with depression [20], higher ACTH levels and a trend to lower response rates to paroxetine treatment [18]. The FKBP5 gene, that encodes for a co-chaperone of heat shock protein 90 that regulates GR sensitivity, has three polymorphisms associated with rapid response to antidepressant treatment [15]. One of them, rs1360780 SNP was associated with higher intracellular FKBP51 protein expression, antidepressant response and higher recurrence of depressive episodes in the lifetime [15]. Also, three polymorphisms in the CRHR1 gene (rs1876828, rs242939, and rs242941) were associated with major depressive disorder and with antidepressant response to FLX in Chinese patients [16,21], and better antidepressant response in a high anxiety depressed group of Mexican-Americans [22]. CRHR2 functioning has been related to reactivity of the HPA axis [23,24], and carriers of the G allele of rs2270007 polymorphism showed worse overall response to citalopram (SSRI) [17]. Finally, as far as we know there are no previous association studies of the AVP gene with MDD or antidepressant response; however, animal model studies suggest that the AVP gene represents a strong candidate to explain the genetic influence in MDD and response to therapy. For instance, AVP overexpression was observed in the paraventricular nucleus of the hypothalamus (PVN) of rat models with extreme anxiety and in stressed or depressed rats [25,26]. AVP overexpression in the extreme anxiety model was caused by a SNP A (−1276) G in the promoter of the AVP gene, reducing the binding of a transcriptional repressor [27,28]. Interestingly, chronic FLX treatment significantly reduced *in vitro* AVP release from rat hypothalamic organ culture [29]. In humans, polymorphisms like the one mentioned in rats have not been described, however polymorphisms in this gene may have subtle effects contributing to related phenotypes. In a previous study (data not shown) we explored for polymorphisms by DNA sequencing of 1.1 kilobases (kb) of the promoter region of the AVP gene (−1050 – +60 bp) in 26 samples of Chilean depressed patients. We found that the rs3729965 SNP was relatively polymorphic and putatively included in a site recognized by a transcription factor (MZF1).

In this article we analyze the relation between circadian salivary cortisol levels and cortisol levels after dexamethasone suppression test (DST) with the placebo response after one week of treatment, with the response to FLX after three weeks and two months of treatment, with remission after two months and with adherence to treatment. Lower levels of salivary cortisol were observed in placebo responders, in FLX responders after two months of treatment and in those that reached full remission than in those who did not respond in each group. Also, a trend to lower cortisol levels was observed in the group of patients who abandoned the study before the third week of treatment compared to the group that continued the treatment. The genetic profiles of seven polymorphisms located in corticotropin releasing hormone receptor 1 (CRHR1, rs242939, rs242941, rs1876828), corticotropin releasing hormone receptor 2 (CRHR2, rs2270007), GR (rs41423247), FK506-binding protein 5 (FKBP5, rs1360780) and Arginine vasopressin (AVP, rs3729965) genes were analyzed in all subjects. This group of polymorphisms does not represent the total genetic variation in these genes.

Two of these SNPs were not in Hardy-Weinberg equilibrium, rs242939 of the CRHR1 gene and rs2270007 of the CRHR2 gene, and only one polymorphism was associated with the early response to FLX (three weeks); rs242939 of CRHR1.

## Methods

### Subjects

This is a prospective longitudinal study that involves clinical follow-up of depressed patients. All examinations were performed according to the tenets of the Declaration of Helsinki. Patients were enrolled in the waiting rooms of two primary health care centers of Santiago, CESFAM Pablo Neruda and CESFAM Juan Antonio Rios, and they were treated as outpatients throughout the entire course of the study. All patients signed a full written informed consent approved by the ethics committee of the Faculty of Medicine of the University of Chile.

To identify patients with current major depressive disorder a two-stage screening process was used. Briefly, all eligible and consenting patients were asked to complete the general health questionnaire (GHQ-12). This brief 12-item instrument, previously validated in Chile, measures current mental health [30-32]. To diagnose major depressive disorder (MDD) and to exclude other psychiatric disorders, individuals with scores of 4 or more in the GHQ-12 were invited for a DSM-IV based Mini International Neuropsychiatric Interview (M.I.N.I.) [33] [American Psychiatric Association, 1994]. The ratings of symptom severity were evaluated using the 17-item version of the Hamilton rating scale for depression (HAM-D_17_) [34]. Three clinicians with formal training in the use of these instruments were calibrated on scale scoring of HAM-D_17_ and MINI. Patients with a score of at least 15 on HAM-D_17_ were included [34].

Exclusion criteria included medical or neurological illnesses, acute or chronic infections, abnormal thyroid function, hypertension, pregnancy, breastfeeding, current substance abuse and co-morbid current psychiatric disorder (psychosis, schizophrenia, generalized anxiety disorders, panic disorders, obsessive compulsive disorders, bipolar depression, severe cognitive impairment and clear suicide risk). The subjects had to be medication-free for at least two months prior to the beginning of the treatment. Also, patients with history of treatment-resistant MDD, defined as non-responders to two previous trials of antidepressants at adequate dosages, were excluded.

This study included the placebo treatment of all patients (lactose tablets) during one week, after which the HAM-D_17_ test was applied again. The placebo response was defined as a reduction in at least 25% of the initial score and to an endpoint lower than 15. We selected this cutoff, which is not very strict, because the placebo treatment was performed during only one week. Greater placebo effects are usually observed after 2–3 weeks of placebo treatment [35], however for ethical reasons and since there are proven treatments for major depression we could not delay the initiation of treatment with FLX any longer. Subsequently, patients were treated with FLX antidepressant only at a dose of 20 mg/day during three weeks, and then increased to 40 mg/day depending on clinical outcome and tolerance. In case of anxiety symptoms or insomnia, alprazolam or zolpidem were prescribed (13% of the patients). To rate changes in the severity of depression and to evaluate the response, assessments using the HAM-D_17_ scale were made at the third week and after two months of FLX treatment. For the purposes of this study the final evaluation was carried out after two months of treatment, although patients continued with it. The therapeutic response was evaluated by calculating the percentages of HAM-D_17_ score reduction by the third week and two months of FLX treatment, ((baseline score – three weeks or two months score) × 100/baseline score). The baseline considered to evaluate response rate to FLX was the HAM-D_17_ score obtained after placebo treatment. Early responders and responders to therapy were defined as those patients showing a reduction in the initial HAM-D_17_ scores of at least 50% after three and eight weeks of treatment, respectively. Remitters were defined as patients with ≤ 7 in HAM-D_17_ after two months of treatment. Non-responders and non-remitters were those who did not reduce the score by at least 50% or to ≤ 7 in the HAM-D_17_ by the corresponding time of treatment. Patients who did not respond to FLX were changed to another antidepressant drug, according to regular clinical practice. To minimize the placebo effect achieved by the professional towards the patient, contacts between patient and physician were established only during scheduled clinical evaluations.

### Endocrine evaluation

After the diagnosis and evaluation of severity using HAM-D_17,_ TSH, free T4 and circadian salivary cortisol levels were evaluated. Patients with altered thyroid hormones were excluded. Normal range for TSH was 0.70-5.52 mUI/L (our results 0.75-5.5) and T4F 0.80-1.80 ng/dL (our results 0.84-1.69) [36,37]. Salivary cortisol levels were evaluated at 08:00, 12:00, 15:00 (after lunch) and 23:00. After the last saliva collection, patients took a low dose of Dexamethasone (0.5 mg) and a new saliva sample was collected at 8:00 AM the following morning. This was performed to evaluate the suppression effect of dexamethasone (dexamethasone suppression test, DST) [38]. The salivary circadian cortisol levels and DST evaluations were performed at the end of the week of placebo and after two months of FLX treatments.

We evaluated the salivary cortisol circadian rhythm by collecting saliva samples in plastic disposable tubes at 08:00, 12:00, 15:00, and 23:00 as previously described [38]. The sensitivity of HPA negative feedback was assessed by the dexamethasone suppression test (DST) [20,21]. For the DST, 0.5 mg of dexamethasone was given at 23:00 and a salivary sample was taken the next day at 08:00. Samples were centrifuged at 1000 × g for 2 min and the free cortisol was measured in the supernatant using the DIASource enzyme immunoassay, (Diasource, Nivelles, Belgium), with a sensitivity of 0.01 μg/dL and intra- and inter-assay coefficients of variation (CV) lower than 10% [38]. The analyses were performed including and excluding patients using oral contraceptives (OC). Subjects with postdexamethasone cortisol levels >1.8 μg/dL were considered non-suppressors [39].

### Genotyping

Five ml of blood were collected in tubes containing EDTA and total DNA was prepared from peripheral blood lymphocytes using the method described by Lahiri & Nurnberger [40].

Genotyping of SNPs located in the CRHR1 (MIM 122561), CRHR2 (MIM 602034), GR (MIM138040), and FKBP5 (MIM 602623) genes was performed by developing PCR-RFLP strategies using the primers and conditions detailed in Additional file 1: Table S1. Briefly, the DNA regions that include polymorphic sites were amplified in 20 μl PCR reactions containing 100 ng genomic DNA, 0.2 mM dNTP, 0.5 pmol/μl of each primer and 1 unit of GoTaq polymerase (Promega, Madison, WI, USA). The temperature profiles included initial denaturation at 95°C for 5 min, followed by 37 cycles of denaturation at 95°C for 30s, annealing at the temperatures indicated in Additional file 1: Table S1 for 30 s, extension at 72°C for 60, and one step of final extension at 72°C for 5 min. The amplicon sizes are listed in the same table. After digestion with restriction enzymes at the appropriate temperatures, the different alleles were defined by electrophoresis in 3% agarose gels.

### Statistical analyses

The HAM-D_17_ results are expressed as mean ± SD. The differences between the cortisol circadian time–course curves of the placebo responders and non-responders, between FLX early responders (after three weeks of treatment) and non-early responders, FLX responders (after two months of treatment) and non-responders, and remitters (after two months of treatment) and non-remitters were determined using t-tests, ANOVA and the general linear model for repeated measures, where the vector of repeated measures of cortisol was considered as a dependent variable and the outcome was considered as an independent variable (placebo response/placebo non-response, FLX response/FLX non-response and remission to FLX/non-remission to FLX). This was performed using SPSS version 15 (SPSS Inc., Chicago, IL). p values less than 0.05 were considered to be statistically significant. The graphs were built using R3.0.2. The statistical power obtained range from 0.79 to 0.99 in the different analyses.

The allele frequencies in responders and non-responders were determined by direct counting. Hardy-Weinberg equilibrium was tested for each marker by comparing the observed and expected genotypes using the χ^2^ or Fisher’s exact test. The relative risks and 95% confidence interval were estimated by Cornfield’s method using the Epi Info program version 6.0 [41].

The linkage disequilibrium (LD), haplotype frequency and association analyses were performed using SNPstats (http://bioinfo.iconcologia.net/SNPstats).

## Results

### Patients and clinical follow-up

**Figure 1:**
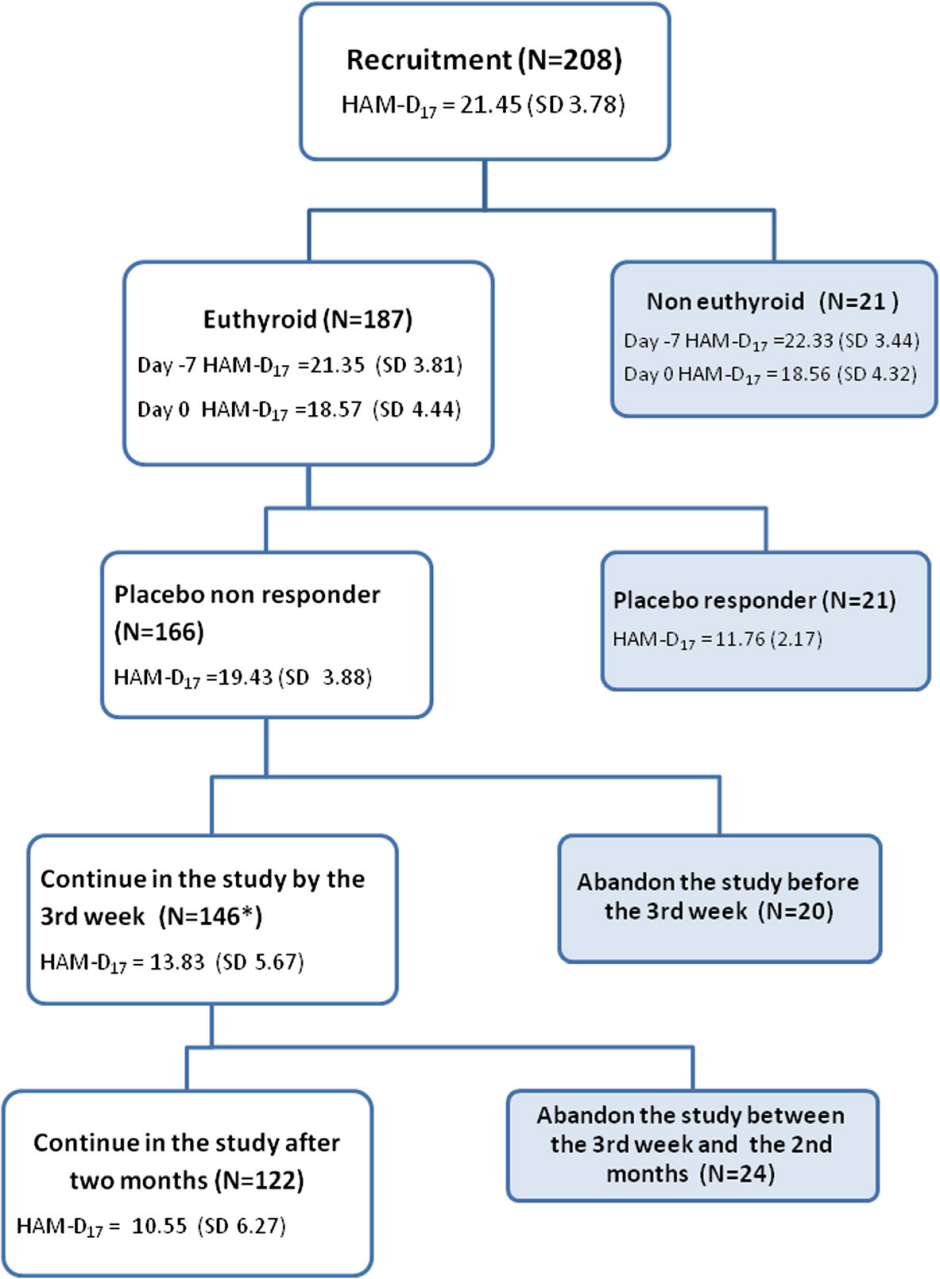
**Diagram illustrating the flow of depressed patients through the study.** The chart shows the recruitment of 208 patients, the exclusion of 21 non-euthyroid individuals, the placebo response, the desertion by the third week and two months and the HAM-D_17_ average scores of each group. *one of the placebo non-responder patients missed this control and thus there are really 146 people still in the study but only 145 evaluated for early response.

Two hundred and eight patients, 7 males and 201 females, ages between 18 and 64 with an average of 43.05 years (Table 1) were diagnosed with MDD (Figure 1). This study included patients with moderate to very severe MDD (HAM-D_17_ total score =15 to 35) with an average of 21.45 (SD = 3.78; CI 20.93-21.96). 90 patients (43.27%) had received previous antidepressant treatment, although none of them had received any antidepressant drug or mood stabilizer in two months prior to the beginning of this study. Most of the participants were housewives (52%) and dependent (20.7%) and independent (18.8%) workers (Table 1).

**Table 1:** **Demographic data of depressed patients**

**Age mean (SD)**	**43.05 (11.21)**
Gender Female	201
Male	7
HAM-D_17_ All patients before placebo treatment (N = 208)	21.45 (SD = 3.78)
Activity	
Housewives	108 (52%)
Dependent worker	43 (20.7%)
Independent worker	39 (18.8%)
Student	4 (1.9%)
Unemployed	11 (5.2%)
other	3 (1.4%)
Non-euthyroid	21 (one male and 20 female; 10.1% of the total)
Oral contraceptive use (OCU)	34 (of the 181 of the euthyroid women)

Thyroid and HPA axis functioning were evaluated in all patients and patients with altered thyroid hormones were excluded. 187 out of the 208 patients were euthyroid (TSH between 0.75-5.5 mUI/L and free-T4 1.84-1.69 ng/dL) and 21 were non-euthyroid. 17 patients were hypothyroid (8.17%) and four hyperthyroid (1.92%), which in total represent 10.1% of the depressed patients. Additionally, circadian salivary cortisol levels and DST were assessed in the 187 euthyroid patients at the end of the week of placebo treatment and again after two months of FLX treatment.

Euthyroid patients (N = 187) were treated with placebo during one week followed by treatment with FLX (20 mg) for two months. Clinical evaluations were performed before and after placebo treatment, after three weeks and again after two months of FLX antidepressant treatment. During the clinical evaluation performed at the third week of FLX treatment (20 mg) the FLX doses were maintained or adjusted to 40 mg depending on the outcome in the patients that remained in the study.

Early response to FLX treatment was defined as a reduction ≥50% of the baseline HAM-D_17_ score after three weeks of treatment (37 out of 145, one patient missed this control and thus there are really 146 people still in the study but only 145 evaluated for early response), response when the reduction was observed after two months (67 out of 122) and remission with reduction to ≤ 7 in the HAM-D_17_ after two months of treatment (48 out of 122) (Table 2). The baseline was the HAM-D_17_ score obtained after placebo treatment.

**Table 2:** **Outcome of depressed patients treated with Fluoxetine**

**Parameter**	**N**	**Outcome**	
		**Yes**	**No**
Early response (week 3)	145*	37 (25.5)	108 (74.5)
Response (by week 8)	122	67 (54.9)	55 (45.1)
Remission (by two months)	122	48 (39.3)	74 (60.6)

Number of subjects and percentages (in parentheses) according to the outcome at the third week and two months of treatment, respectively. *one patient missed this control and thus there are really 146 people still in the study but only 145 evaluated for early response.

After one week of placebo treatment the clinical evaluation of the entire group of patients by means of HAM-D_17_ showed a reduction of the scores to an average of 18.57 (SD = 4.44; CI 17.93-19.21). Only six patients (3.2%) showed the placebo response, defined as a reduction in HAM-D_17_ scores by at least 50%, two of whom reached full remission after two months of FLX treatment. Since the placebo treatment period was too short, only one week, we decided to apply less strict criteria to classify placebo responders. Therefore the cutoff for placebo response was defined as a reduction of at least 25% of the baseline and scoring less than 15 in the HAM-D_17_ scale. Under this criterion 21 out of the 187 patients (11.23%) responded to placebo. Placebo responders started with HAM-D_17_ average scores of 21.67 (SD = 3.02; CI: 20.29-23.04) and reduced it significantly (p < 0.0001) to an average of 11.76 (SD = 2.17; CI: 10.78-12.75). The placebo non-responder group started with a very similar HAM-D_17_ average score of 21.31 (SD = 3.90; CI: 20.71-21.9) and reduced it to an average of 19.43 by the end of the placebo trial (SD = 3.88; CI: 18.83-20.02). The reduction in this non-responder group was also significant (p < 0.0001), although it represented only a score reduction of 8.8%. The initial HAM-D_17_ average scores before the initiation of placebo treatment were not significantly different in the placebo responder and non-responder groups (21.67 compared to 21.31); 82 out of the 187 euthyroid patients (43.85%) had received previous antidepressant treatment. Only four of them (4.88%) responded to placebo while the remaining 78 did not (95.22%). In the group of 105 patients with no previous antidepressant treatment, 17 responded to placebo (16.2%) and 88 did not (83.8%). Therefore, prior antidepressant treatments and consequently prior depressive episodes were actually related to worse placebo response outcome (p = 0.019, calculated by the Fisher exact test).

After the placebo trial, the euthyroid patients who did not respond to placebo exhibited a HAM-D_17_ average score of 19.43 (SD = 3.88; CI: 18.83-20.02) (Figure 1). For our purposes we excluded placebo responders from the analyses, although they continued with the treatment, thus the FLX study began with 166 patients (Figure 1). Twenty patients (12.65%) abandoned the study between the initiation and the third week of therapy, and 24 more patients (13.86%) did so between the third week and two months. The H AM-D_17_ total average scores of the patients that continued the treatment reduced to 13.83 (SD = 5.67; CI 12.81-14.86) after three weeks of treatment and to 10.55 (SD = 6.27; CI 9.42-11.67) after two months (Figure 1).

Only 16 out of 78 patients who had received previous antidepressant treatment and who did not respond to placebo responded by the third week of FLX treatment (20.51%); 31 patients did after two months (39.74%) and 22 reached full remission (28.2%). We did not find any relation between the early responses, response or remission rates with previous antidepressant treatments.

There were 42 out of the total 187 euthyroid patients with stressor o trauma history, 7 of whom responded to placebo and 9 were early responders, although we did not find any association between trauma and placebo or early response to FLX. Of the 67 patients who responded to FLX after two months of treatment 10 had stressor or trauma history (14.93%), and 16 of the 55 who did not respond to FLX (29.09%) had stressor or trauma history. These results approach statistical significance (p = 0.057, calculated by chi-square test).

Similarly, of the 48 patients who remitted with FLX, 6 had stressor or trauma history (12.5%) and 20 of the 74 that did not remit with FLX had stressor or trauma history (27.03%). This difference was almost significant and the trauma history could be related to poorer response (p = 0.056, calculated by chi-square test).

There were 11 patients using benzodiazepines who completed two months of FLX trial. One of these patients remitted (9.09%) and 10 did not (90.91%). In the group of patients not using benzodiazepines (N = 111) 47 remitted (41.82%) and 64 did not (58.18%). Benzodiazepine use was significantly associated with remission (p = 0.049, calculated by the Fisher exact test). There was no relationship between benzodiazepine use and placebo, FLX early (3 weeks) or late (two months) response (data not shown).

### Relationship between salivary cortisol levels and DST to placebo and FLX response/remission

**Figure 2:**
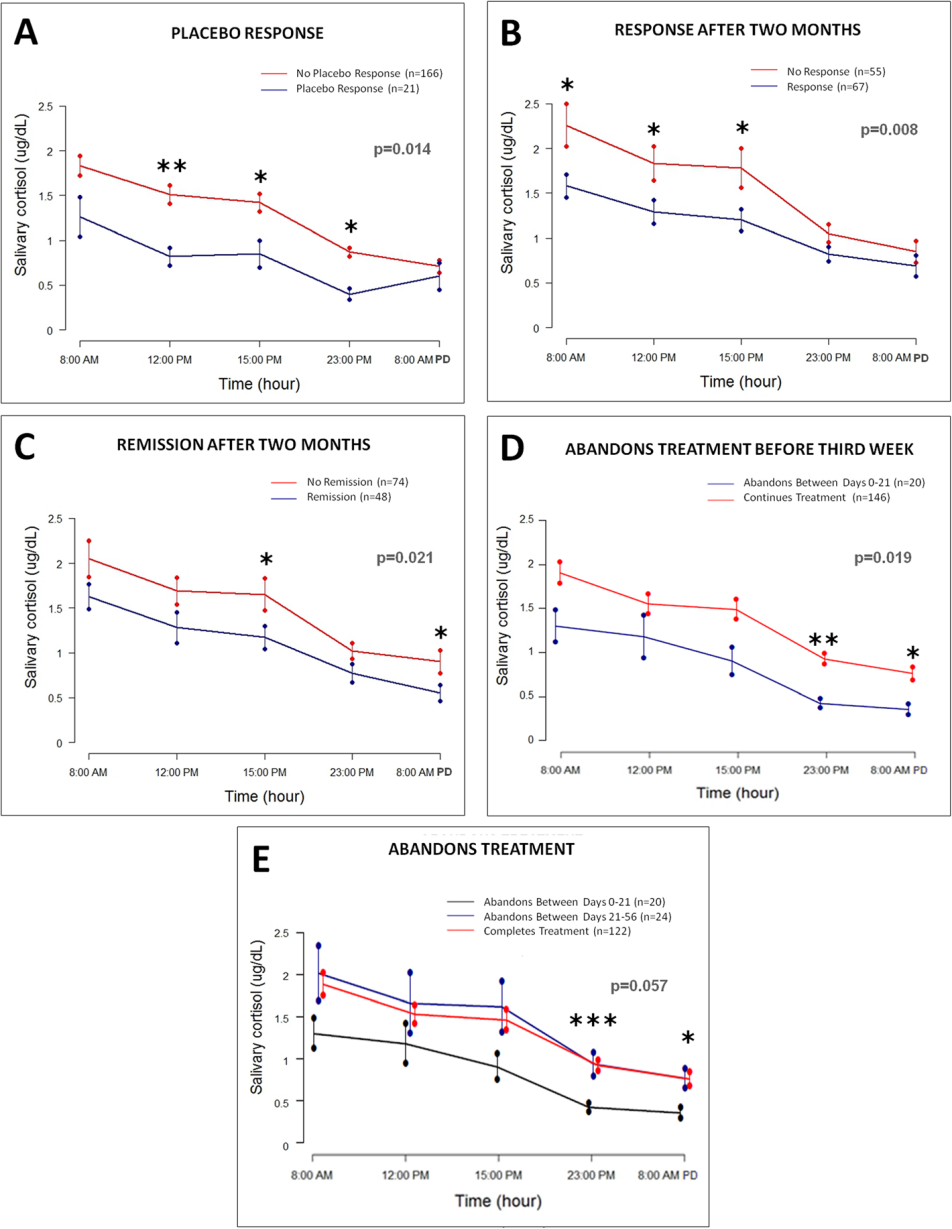
**Baseline circadian salivary cortisol levels and cortisol levels after DST.** Differences in baseline circadian salivary cortisol levels between: placebo responders and non-responders **(A)**, FLX responders and non-responders **(B)**, FLX remitters and non-remitters **(C)**, and between the groups that abandoned the treatment before and after the third week of FLX treatment and the group that completed the two months **(D)** and comparing the group that completed the treatment to those who abandoned after the third week **(E)**. The colors of the curves are described in each graph. The asterisks represent statistical significance evaluated by *t*-test (* = p < 0.05 and ** p <0.01) except in the case of the abandonment study which was assessed by ANOVA (Kruskal-Wallis). The graphs also show the p values obtained by comparing the curves using the general linear model for repeated measures. Error bars represent the SEM.

Salivary cortisol rhythm in MDD patients was determined at the end of the week of placebo treatment (baseline) and after two months of FLX treatment. As expected, salivary cortisol levels were high in the morning and declined throughout the day (Figure 2). Baseline cortisol levels were compared among the groups that responded and those who did not respond to placebo treatment. Significant differences were found, with lower circadian salivary cortisol levels in the group that responded (p = 0.014; calculated by the general linear model for repeated measures) (Figure 2A). These differences were significant at 12:00 (p = 0.000008), 15:00 (p = 0.003) and 23:00 (p = 0.0028).

Baseline salivary cortisol levels were not significantly different between early and non-early responders (p = 0.53). Nevertheless, in the group of responders after two months of treatment there was a significant difference in baseline salivary cortisol levels compared to non-responders (p = 0.008, calculated by the general linear model for repeated measures) (Figure 2B). These differences were significant at 8:00 (p = 0.016), 12:00 (p = 0.022), and 15:00 (p = 0.024).

Something similar occurred with the remitters (N = 48), whose baseline salivary cortisol levels were significantly lower than those of non-remitters (p = 0.021) (Figure 2C). These differences were significant at 15:00 (p = 0.031).and 8:00 (p = 0.048) after DST.

No significant differences were observed between salivary circadian cortisol levels and DST before and after FLX treatment, independently of the antidepressant treatment outcome (p > 0.05).

Since interference of oral contraceptive (OC) use in cortisol measurement has been reported, we performed the same analyses excluding OC users; however the significance of the analysis remained (data not shown).

Additionally 175 patients out of the 187, representing 93.6% of the group of the euthyroid patients suppressed after 0.5 mg of dexamethasone intake. Consequently 12 (6.4%) did not suppress; one responded to placebo, five responded after three weeks of treatment, one abandoned the study, five did not respond at any time and three reached full remission. There were no significant differences in the response or remission rates between the groups that suppressed or not in the DST.

Interestingly, the group that abandoned the treatment before the third week of treatment had significantly lower circadian cortisol levels compared to the patients who continued with treatment (p = 0.019 calculated by general linear model for repeated measures) (Figure 2D). This difference was significant at 23:00 (p = 0.0025, calculated by *t*-test) and after DST (p = 0.0469, calculated by *t*-test) (Figure 2D).

In a more detailed analysis dividing the group that continued the treatment into “abandoned between the third and eighth week” and “completed treatment” the results approached significance (p = 0.057, general linear model for repeated measures) (Figure 2E).

### Genetic associations

We genotyped seven polymorphisms located in the CRHR1, CRHR2, FKBP5, AVP and GR genes (rs1876828, rs242939, rs242941, rs2270007, rs1360780, rs3729965 and rs41423247) (Additional file 1: Table S1). Five polymorphisms (rs242941, rs1876828, rs1360780, rs3729965, rs41423247) were in Hardy Weinberg equilibrium and two were not (rs242939 of CRHR1 gene with p = 0.013 and rs2270007 of the CRHR2 gene with p = 0.018), (Additional file 1: Table S2). We compared the allele frequencies with those reported in the 1000 Genome Project. Most of the Chilean allele frequencies observed were closest to the allele frequencies reported in Mexican population (Additional file 1: Table S2).

Next we evaluated whether the polymorphisms were associated with placebo or FLX treatment outcome (Additional file 1: Tables S3–S6). We did not find any association between placebo response and the seven polymorphisms (Additional file 1: Table S3). In the association study between the outcome after FLX treatment, including early response (three week of FLX treatment), response and remission (two months of FLX treatment) and the same seven polymorphisms we only found association between early response and the polymorphism rs242939 of the CRHR1 gene, comparing genotype by genotype (global p = 0.017), or comparing the two groups using different models (Additional file 1: Table S4).

The SNPs rs242939, rs242941 and rs1876828 of CRHR1 genes were in linkage disequilibrium as expected, with D’ values of 0.9986 between rs242939 and rs242941, 0.9935 between rs242939 and rs1876828 and 0.8961 between rs242941 and rs1876828. The most frequent haplotype was AGG. No significant differences were observed comparing responders and non-responders either to placebo or FLX (data not shown).

We did not find any relation between cortisol levels and any of the polymorphisms.

## Discussion

208 patients with MDD were recruited in primary health care centers of Santiago; most of them (N = 201) were women, while only 7 were men. The gender bias in the recruited patients may be explained by several factors, such as higher prevalence of MDD in women than in men, men not wanting to participate due to work reasons, the interviews being performed during working hours when more homemaker women than men attend medical services, as shown by 52% of our recruited MDD patients being housewives, among others. The low number of males included in this study prevents us from evaluating the role of gender in the outcome. The average of the HAM-D_17_ total score of the recruited patients (21.45, SD 3.78) corresponds to moderate to very severe depression (HAM-D_17_ total score =15 to 35).

The non-euthyroid patients were 10.1% of the total recruited patients; 8.2% were hypothyroid and 1.9% hyperthyroid. This result is in agreement with other studies carried out in similar kinds of patients. For instance, Chilean individuals with anxiety and mood disorders exhibited 9.7% hypothyroidism and 2.2% hyperthyroidism [42]. The prevalence of hypothyroidism in the general population is 1-2%; it is more frequent in women and in the elderly [43]. The high rate of hypothyroidism observed supports the idea that it is more prevalent in patients with mood disorders [42].

### Placebo effect in depressed patients

Placebo effect has been defined as “any improvement of symptoms or signs following a physically inert intervention” [44]. The placebo effect is especially effective in relieving subjective symptoms such as pain, fatigue, anxiety and depression, although the best understood is the one that works on analgesic responses.

For antidepressant treatments, reduction in rating scales either for placebo or different antidepressants is perceived in the first week, however most placebo antidepressant effects are observed after 2–3 weeks [35,45-47]. In our study, although many patients improved after placebo treatment, only six of them reduced at least 50% of the HAM-D_17_ score after placebo. This was expected considering the short period of placebo treatment. Therefore, we reduced the strictness of the placebo response classification to a reduction of at least 25% of HAM-D_17_ scores and to scores lower than 15 by the end of the week. The period we used placebo was limited due to ethical reasons. Under these conditions we found that 11.23% of the patients responded to placebo, which could be considered as a high rate in view of the short period of placebo treatment selected [11].

Placebo effects are influenced by several factors such as caregiver interaction, learned expectations or experience with previous treatments. Caregiver interaction was controlled in this study by limiting the contact between patient and physician to scheduled clinical evaluations, but the other two factors could not be prevented. In fact, our results showed that previous antidepressant treatments, which could be related to expectations and previous experiences, were associated with poorer placebo response (p = 0.019). Thus patients with no previous antidepressant treatment had a better chance of responding to placebo.

### Placebo effect and circadian salivary cortisol levels

The placebo effect is a psychobiological phenomenon that can be attributable to neurobiological mechanisms [48]. Recently, low cortisol levels in depressed patient with early life stress experiences who also responded to placebo treatment were reported, suggesting that the mechanisms of placebo effect could have physiological bases such as HPA axis activity [48]. Something similar was observed in studies of placebo effects in analgesia, with the report of reduction of cortisol levels after placebo administration when preconditioned with sumatripan (a 5-HT1B/1D agonist that stimulates GH and inhibits cortisol secretion) [49]. Moreover, literature reports demonstrated that placebo treatments partially reproduce cerebral glucose metabolism in FLX-treated depressed men in randomized, placebo-controlled studies (Reviewed by Benedetti [48]). We determined circadian salivary cortisol levels and DST, and evaluated their association with placebo treatment outcome. Lower baseline salivary cortisol levels were consistently related to better response to placebo intake (Figure 2), supporting that HPA axis activity could have a role in the mechanisms of placebo antidepressant effect and that placebo responders correspond to a distinct biological group. For example, this group could be composed of people suffering from a DSM-IV adaptive disorder rather than MDD. Additionally, a meta-analysis carried out by Knorr et al. showed higher salivary cortisol levels in depressed patients compared to controls [50]. It has been shown in several studies that cortisol or hydrocosterone may alter autobiographical and emotional perception [51]. Thus, it could be proposed that elevated cortisol may be related to altered perception of events and the course of depression disorder. Similarly, lower cortisol levels in the placebo and FLX responder groups may also indicate lesser biological disturbance in stress regulatory mechanisms.

Nevertheless, in our placebo effect study we evaluated cortisol levels at the end of the week of placebo treatment, therefore we do not know whether the cortisol levels were reduced as a consequence of the placebo treatment or if baseline levels were already low [49]. Further studies could be performed to determine this.

Finally, we cannot discount that the placebo effect observed did have a causal connection with the outcome. This could be explained by other causes such as the natural course of the disease or more desire or motivation for improvement in the group with lower HPA activity [52,53]. Recently, positive expectations of outcome have been associated with better outcomes [12,54,55]. In fact, the antidepressant response observed following drug treatment may include a placebo effect, therefore in our study the relatively low response rate (54%) might be explained by the exclusion of the placebo responders, who would have probably responded to FLX treatment too. Thus placebo responders could most likely to benefit from a biologically active treatment.

### FLX treatment outcome and circadian salivary cortisol levels

Circadian salivary cortisol levels and DST, and their relationship with placebo and FLX treatment outcome were evaluated. Our results show that lower salivary cortisol levels were consistently related to better response to both placebo and FLX after two months of treatment (Figure 2). No significant difference was observed between early and non-early responders. The results were the same when we excluded the OC users. There is evidence that oral contraceptive use results in higher corticosteroid-binding globulin (CBG) levels with consequent higher total cortisol levels. This increase only affects bound cortisol; free cortisol levels are unaltered in states of increased CBG [56]. Therefore the similarity between results using OC or not is reliable.

The relationship between higher cortisol levels and poorer response to placebo and FLX treatments is in agreement with the hypothesis that the extent of dysregulation of HPA might be related to worse outcome. The elevated baseline cortisol levels in non-responders could be explained by several factors such as differential genetic and/or epigenetic individual profiles that maintain cortisol levels elevated.

No differences in cortisol after DST was observed in the groups of responders and non-responders.

Non-suppression of cortisol secretion by dexametasone has been reported in depressed patients, supposedly caused by altered cortisol feedback inhibition [57]. In previous studies we performed DST in depressed patients using the standard dose of 1 mg dexametasone, observing high suppression rates (data not shown). Therefore, in this study we performed the DST using a reduced dose of dexametasone (0.5 mg). Interestingly, 93.6% of the patients suppressed after intake of dexamethasone and consequently only 6.4% of the whole group of euthyroid patients did not exhibit suppression. These differences with other studies may be related to several factors such as ethnic differences and different diagnostic criteria used. The suppression after dexametasone intake was not related to the capacity to respond to placebo or FLX, however in remitters cortisol levels were significantly lower after DST than in non-remitters. This suggests that remitters are more sensitive to cortisol feedback inhibition.

### Genetic associations

Many studies have associated polymorphisms in genes involved in the HPA axis, MDD and other personality traits [58-60] with antidepressant response [60,61]. For instance, some reports have suggested a relationship between CRHR1 polymorphisms (rs1876828, rs242939, and rs242941) and both the risk of suffering major depression [17,21,62] and antidepressant response to FLX [16,22,62]. CRHR2 polymorphisms have been associated with increased risk of suffering major depression with borderline significance [17,58] and with a worse overall response to citalopram (SSRI) [17]. Similar associations have been reported for polymorphisms in the AVP, GR and FKBP5 genes [15,59-61,63].

The CRHR1 (rs242941, rs1876828), GR (rs41423247), FKBP5 (rs1360780) and AVP (rs3729965) polymorphisms analyzed in this study were in Hardy Weinberg equilibrium, however the polymorphisms of rs242939 of CRHR1 and rs2270007 of the CRHR2 genes were not. It is possible that the bias in the sample selection, i.e. depressed patients, may explain these results. If these polymorphisms are related to MDD, then the selected group does not represent the general population, explaining the disequilibrium observed. A comparison with non-depressed subjects of the same population could help to decipher this issue.

In the association analyses between the polymorphisms and the outcome, allele and genotype association with the polymorphism rs242939 of CRHR1 (p = 0.043) was found. These results strengthen the concept that CRHR1 is involved in antidepressant response and also suggest that CRHR1 could have greater effect than other genes whose association was not detected in this study under the conditions used, but have been associated by other groups. CRHR1 is a receptor of CRH with higher affinity for it than CRHR2 and is highly expressed in the hippocampus, cortex and cerebellum [64]. CRHR1 has been related to BDNF expression in the hippocampus. In animal models, the increase in corticoids induced by stress leads to reduction of the apical dendrites of pyramidal neurons of the CA3 region of the hippocampus, an effect related to reduction of BDNF expression (reviewed by Ventura-Juncá [65]). Interestingly, the hippocampal volume reduction could be reversed by antidepressants [66]. The rs242939 polymorphism is located in an intron region of the CRHR1 gene; it has is yet to be determined if the genetic association observed here and by others is caused by a direct functional biological effect of this polymorphism or another linked to it.

The lack of expected association effects of other polymorphisms in CRHR1, CRHR2, GR, FKBP5 and AVP genes could be interpreted in several ways. First of all, MDD is a very complex disorder in which many genes each of small effect could be interacting. If epistatic effects involving some or all of these genes are occurring, much larger sample sizes should be studied. Also, there might be ethnic differences in allele frequencies and/or different linkage groups with other functional polymorphisms in the Chilean population compared to those included in the publications reporting association. In turn, the treatment response might be also influenced by other factors such as epigenetic alterations in genes important to HPA functioning [65,67]. Additionally, the disorder evaluated in this study is moderate to severe depression. Probably, a more extreme phenotype could have shown a more important genetic involvement or a stricter definition of the phenotype might be more related to the genes. Lastly, it is possible that the lack of reproducibility of other studies could be caused by a publication bias effect toward positive results, generating the idea that experiments with negative results rarely occur. Thus genetics might explain only few of the causes of depression and outcome.

Gene-gene interactions have been hypothesized to be related to MDD and to antidepressant outcome. The sample size of our study and the low allele frequency of each minor allele do not allow us to perform these analyses.

### Previous treatment effect on outcome after FLX treatment

As mentioned before, personal history of previous antidepressant treatments was significantly associated with unresponsiveness to placebo (p = 0.019). Nevertheless, it was not related to response to FLX treatment at any time or to remission. This suggests that the widely reported antidepressant unresponsiveness in patients with history of previous ineffective treatments could be more related to reduction in placebo effect rather than to a lack of neurochemical action of antidepressants [68]. This might be related to the partial response reported within 2 weeks of antidepressant treatment as the most important positive predictor for achieving remission [68]. We propose that this quick response could be more related to the placebo effect than to antidepressant specific action(s).

### Adherence to a treatment

26.5% of the patients who initiated FLX treatment abandoned it. Interestingly the group that abandoned the treatment before the third week of FLX treatment may have had lower circadian cortisol levels and cortisol levels after DST (p = 0.057). Nevertheless this group included only 20 patients and the significance is borderline. There might be several reasons to abandon a treatment, including different personalities or mood conditions that may correlate with compliance. Unfortunately, since this group of patients did not return to the clinical evaluation it is not possible to find the actual reasons. One possible explanation could be a very rapid effect of the antidepressant treatment and the feeling of the patients that they do not need any further treatment or medical supervision. This option could be related to lower or no HPA dysregulation, expressed as lower cortisol levels. On the contrary, the abandonment could be related to no response, persistence of depressive symptoms and disappointment with the treatment, adverse effects of FLX or to the placebo, etc. None of these possibilities are likely related to lower cortisol levels. More studies must be performed to confirm this data and to explore the factors affecting adherence.

## Conclusions

Our data confirm the relevance of cortisol levels in the response of depressed patients either to placebo or FLX treatment; however the genetic data only supports the association of rs242939 polymorphism of CRHR1 with the response but does not support association with other polymorphisms reported in the literature. These results suggest that there may be other factors involved in antidepressant response, such as polymorphisms with very low effect probably interacting with other factors.

Further insights into the mechanisms of response to placebo and to medications are needed. Identification of biomarkers, genetic or otherwise, that can help to predict antidepressant response would be of great clinical relevance.

## Additional file

Additional file 1:
**Genotyping, Hardy Weinberg equilibrium and association analyses.** The additional file is an Excel file with three worksheets. The first sheet **(Table S1)** includes the information of the primer sequences, PCR conditions used in the genotyping and the product lengths obtained after enzymatic digestion. The second **(Table S2)** contains the genotype and allele frequencies obtained, the results of Hardy Weinberg equilibrium evaluation in the recruited patients and the comparison of the allele frequencies to other populations. The last worksheet consists of four tables **(Tables S3–S6)** containing the associations between the seven polymorphisms genotyped with placebo response **(Table S3)**, early response to FLX **(Table S4)**, response after two months of FLX treatment **(Table S5)** and remission **(Table S6)**.

## Abbreviations

ACTH: Adrenocorticotropic hormone
AVP: Arginine vasopressin
CRHR1: Corticotropin releasing hormone receptor 1
CRHR2: Corticotropin releasing hormone receptor 2
DST: Dexamethasone suppression test
FKBP5: FK506-binding protein 5
FLX: Fluoxetine
GHQ-12: General health questionnaire
GR: Glucocorticoid receptor
HAM-D_17_: Hamilton depression scale
HPA: Hypothalamic-pituitary-adrenal
MDD: Major depressive disorder
M.I.N.I: Mini international neuropsychiatric interview
OC: Oral contraceptive
SSRIs: Selective serotonin reuptake inhibitors
PVN: Paraventricular nucleus of the hypothalamus
Kb: Kilobases

## References

[1] Vicente B, Kohn R, Rioseco P, Saldivia S, Levav I, Torres S (2006). Lifetime and 12-month prevalence of DSM-III-R disorders in the Chile psychiatric prevalence study. Am J Psychiatry.

[2] Kendler KS, Thornton LM, Gardner CO (2000). Stressful life events and previous episodes in the etiology of major depression in women: an evaluation of the “kindling” hypothesis. Am J Psychiatry.

[3] Papakostas GI, Thase ME, Fava M, Nelson JC, Shelton RC (2007). Are antidepressant drugs that combine serotonergic and noradrenergic mechanisms of action more effective than the selective serotonin reuptake inhibitors in treating major depressive disorder? A meta-analysis of studies of newer agents. Biol Psychiatry.

[4] Gartlehner G, Chapman A, Strobelberger M, Thaler K (2010). Differences in efficacy and safety of pharmaceutical treatments between men and women: an umbrella review. PLoS One.

[5] Anacker C, Zunszain PA, Carvalho LA, Pariante CM (2011). The glucocorticoid receptor: pivot of depression and of antidepressant treatment?. Psychoneuroendocrinology.

[6] Horstmann S, Binder EB (2011). Glucocorticoids as predictors of treatment response in depression. Harv Rev Psychiatry.

[7] Wong ML, Licinio J (2001). Research and treatment approaches to depression. Nat Rev Neurosci.

[8] Fernández-Guasti AF, Herrera L, Handa R (2012). Sex, stress and mood disorders: at the intersection of adrenal and gonadal hormones. Horm Metab Res.

[9] Varghese FP, Brown ES (2001). The hypothalamic-pituitary-adrenal axis in major depressive disorder: a brief primer for primary care physicians. Prim Care Companion J Clin Psychiatry.

[10] Ribeiro SC, Tandon R, Grunhaus L, Greden JF (1993). The DST as a predictor of outcome in depression: a meta-analysis. Am J Psychiatry.

[11] Kirsch I (2008). Challenging received wisdom: antidepressants and the placebo effect. McGill J Med.

[12] Mayberg HS, Silva JA, Brannan SK, Tekell JL, Mahurin RK, McGinnis S, Jerabek PA (2002). The functional neuroanatomy of the placebo effect. Am J Psychiatry.

[13] Vallance AK (2007). A systematic review comparing the functional neuroanatomy of patients with depression who respond to placebo to those who recover spontaneously: is there a biological basis for the placebo effect in depression?. J Affect Disord.

[14] Baes C, Martins CM, Tofoli SM, Juruena MF (2014). Early life stress in depressive patients: HPA axis response to GR and MR Agonist. Front Psychiatry.

[15] Binder EB, Salyakina D, Lichtner P, Wochnik GM, Ising M, Putz B, Papiol S, Seaman S, Lucae S, Kohli MA, Nickel T, Kunzel HE, Fuchs B, Majer M, Pfennig A, Kern N, Brunner J, Modell S, Baghai T, Deiml T, Zill P, Bondy B, Rupprecht R, Messer T, Kohnlein O, Dabitz H, Bruckl T, Muller N, Pfister H, Lieb R (2004). Polymorphisms in FKBP5 are associated with increased recurrence of depressive episodes and rapid response to antidepressant treatment. Nat Genet.

[16] Liu Z, Zhu F, Wang G, Xiao Z, Tang J, Liu W, Wang H, Liu H, Wang X, Wu Y, Cao Z, Li W (2007). Association study of corticotropin-releasing hormone receptor1 gene polymorphisms and antidepressant response in major depressive disorders. Neurosci Lett.

[17] Papiol S, Arias B, Gasto C, Gutierrez B, Catalan R, Fananas L (2007). Genetic variability at HPA axis in major depression and clinical response to antidepressant treatment. J Affect Disord.

[18] Brouwer JP, Appelhof BC, van Rossum EF, Koper JW, Fliers E, Huyser J, Schene AH, Tijssen JG, Van Dyck R, Lamberts SW, Wiersinga WM, Hoogendijk WJ (2006). Prediction of treatment response by HPA-axis and glucocorticoid receptor polymorphisms in major depression. Psychoneuroendocrinology.

[19] van Rossum EF, Binder EB, Majer M, Koper JW, Ising M, Modell S, Salyakina D, Lamberts SW, Holsboer F (2006). Polymorphisms of the glucocorticoid receptor gene and major depression. Biol Psychiatry.

[20] Galecka E, Szemraj J, Bienkiewicz M, Majsterek I, Przybylowska-Sygut K, Galecki P, Lewinski A (2013). Single nucleotide polymorphisms of NR3C1 gene and recurrent depressive disorder in population of Poland. Mol Biol Rep.

[21] Liu Z, Zhu F, Wang G, Xiao Z, Wang H, Tang J, Wang X, Qiu D, Liu W, Cao Z, Li W (2006). Association of corticotropin-releasing hormone receptor1 gene SNP and haplotype with major depression. Neurosci Lett.

[22] Licinio J, O’Kirwan F, Irizarry K, Merriman B, Thakur S, Jepson R, Lake S, Tantisira KG, Weiss ST, Wong ML (2004). Association of a corticotropin-releasing hormone receptor 1 haplotype and antidepressant treatment response in Mexican-Americans. Mol Psychiatry.

[23] Bale TL, Contarino A, Smith GW, Chan R, Gold LH, Sawchenko PE, Koob GF, Vale WW, Lee KF (2000). Mice deficient for corticotropin-releasing hormone receptor-2 display anxiety-like behaviour and are hypersensitive to stress. Nat Genet.

[24] Coste SC, Kesterson RA, Heldwein KA, Stevens SL, Heard AD, Hollis JH, Murray SE, Hill JK, Pantely GA, Hohimer AR, Hatton DC, Phillips TJ, Finn DA, Low MJ, Rittenberg MB, Stenzel P, Stenzel-Poore MP (2000). Abnormal adaptations to stress and impaired cardiovascular function in mice lacking corticotropin-releasing hormone receptor-2. Nat Genet.

[25] Wotjak CT, Ludwig M, Ebner K, Russell JA, Singewald N, Landgraf R, Engelmann M (2002). Vasopressin from hypothalamic magnocellular neurons has opposite actions at the adenohypophysis and in the supraoptic nucleus on ACTH secretion. Eur J Neurosci.

[26] Nakase S, Kitayama I, Soya H, Hamanaka K, Nomura J (1998). Increased expression of magnocellular arginine vasopressin mRNA in paraventricular nucleus of stress-induced depression-model rats. Life Sci.

[27] Murgatroyd C, Wigger A, Frank E, Singewald N, Bunck M, Holsboer F, Landgraf R, Spengler D (2004). Impaired repression at a vasopressin promoter polymorphism underlies overexpression of vasopressin in a rat model of trait anxiety. J Neurosci.

[28] Landgraf R, Kessler MS, Bunck M, Murgatroyd C, Spengler D, Zimbelmann M, Nussbaumer M, Czibere L, Turck CW, Singewald N, Rujescu D, Frank E (2007). Candidate genes of anxiety-related behavior in HAB/LAB rats and mice: focus on vasopressin and glyoxalase-I. Neurosci Biobehav Rev.

[29] Altemus M, Cizza G, Gold PW (1992). Chronic fluoxetine treatment reduces hypothalamic vasopressin secretion *in vitro*. Brain Res.

[30] Araya R, Wynn R, Lewis G (1992). Comparison of two self administered psychiatric questionnaires (GHQ-12 and SRQ-20) in primary care in Chile. Soc Psychiatry Psychiatr Epidemiol.

[31] Cabana MD, Rand CS, Powe NR, Wu AW, Wilson MH, Abboud PA, Rubin HR (1999). Why don’t physicians follow clinical practice guidelines? A framework for improvement. JAMA.

[32] Von Korff M, Shapiro S, Burke JD, Teitlebaum M, Skinner EA, German P, Turner RW, Klein L, Burns B (1987). Anxiety and depression in a primary care clinic. Comparison of diagnostic interview schedule, general health questionnaire, and practitioner assessments. Arch Gen Psychiatry.

[33] Sheehan DVLY, Harnett-Sheehan K, Amorim P, Janavs J, Weiller EHT, Baker R, Dunbar G (1998). The Mini-International Neuropsychiatric Interview (M.I.N.I.): the development and validation of a structured diagnostic psychiatric interviewfor DSMIV and ICD-10. J Clin Psychiatry.

[34] Hamilton M (1960). A rating scale for depression. J Neurol Neurosurg Psychiatry.

[35] Stassen HH, Angst J, Hell D, Scharfetter C, Szegedi A (2007). Is there a common resilience mechanism underlying antidepressant drug response? Evidence from 2848 patients. J Clin Psychiatry.

[36] Mosso L, Margozzini P, Trejo P, Dominguez A, Solari S, Valdivia G, Arteaga E (2013). Thyroid stimulating hormone reference values derived from the 2009–2010 Chilean National Health Survey. Rev Med Chil.

[37] Peralta Watt M, Penalver Talavera D (2008). Panhypopituitarism due to craniopharyngioma associated with hyperthyroidism caused by graves’ disease. Endocrinol Nutr.

[38] Araya AV, Rojas P, Fritsch R, Rojas R, Herrera L, Rojas G, Gatica H, Silva H, Fiedler JL (2006). Early response to venlafaxine antidepressant correlates with lower ACTH levels prior to pharmacological treatment. Endocrine.

[39] Stewart PM KN, Kronenberg HM, Melmed S, Polonsky KS, Larsen PR (2011). The Adrenal Cortex. Williams Textbook of Endocrinology.

[40] Lahiri DK, Nurnberger JI (1991). A rapid non-enzymatic method for the preparation of HMW DNA from blood for RFLP studies. Nucleic Acids Res.

[41] Cornfield J (1956). A Statistical Problem Arising From Retrospective Studies., vol. Volume IV, (Negman, J. ed.).

[42] Fardella C, Gloger S, Figueroa R, Santis R, Gajardo C, Salgado C, Barroilhet S, Foradori A (2000). High prevalence of thyroid abnormalities in a Chilean psychiatric outpatient population. J Endocrinol Invest.

[43] Vanderpump MP (2011). The epidemiology of thyroid disease. Br Med Bull.

[44] Tavel ME (2014). The placebo effect: the good, the bad and the ugly. Am J Med.

[45] Rutherford BR, Roose SP (2013). A model of placebo response in antidepressant clinical trials. Am J Psychiatry.

[46] Rutherford BR, Sneed JR, Roose SP (2009). Does study design influence outcome? The effects of placebo control and treatment duration in antidepressant trials. Psychother Psychosom.

[47] Walsh BT, Seidman SN, Sysko R, Gould M (2002). Placebo response in studies of major depression: variable, substantial, and growing. JAMA.

[48] Benedetti F, Mayberg HS, Wager TD, Stohler CS, Zubieta JK (2005). Neurobiological mechanisms of the placebo effect. J Neurosci.

[49] Benedetti F, Pollo A, Lopiano L, Lanotte M, Vighetti S, Rainero I (2003). Conscious expectation and unconscious conditioning in analgesic, motor, and hormonal placebo/nocebo responses. J Neurosci.

[50] Knorr U, Vinberg M, Kessing LV, Wetterslev J (2010). Salivary cortisol in depressed patients versus control persons: a systematic review and meta-analysis. Psychoneuroendocrinology.

[51] Buchanan TW, Lovallo WR (2001). Enhanced memory for emotional material following stress-level cortisol treatment in humans. Psychoneuroendocrinology.

[52] Oken BS (2008). Placebo effects: clinical aspects and neurobiology. Brain.

[53] Miller FG, Kaptchuk TJ (2008). The power of context: reconceptualizing the placebo effect. J R Soc Med.

[54] Mondloch MV, Cole DC, Frank JW (2001). Does how you do depend on how you think you’ll do? A systematic review of the evidence for a relation between patients’ recovery expectations and health outcomes. CMAJ.

[55] Volkow ND, Wang GJ, Ma Y, Fowler JS, Zhu W, Maynard L, Telang F, Vaska P, Ding YS, Wong C, Swanson JM (2003). Expectation enhances the regional brain metabolic and the reinforcing effects of stimulants in cocaine abusers. J Neurosci.

[56] Kirschbaum C, Hellhammer DH (1994). Salivary cortisol in psychoneuroendocrine research: recent developments and applications. Psychoneuroendocrinology.

[57] Pariante CM, Miller AH (2001). Glucocorticoid receptors in major depression: relevance to pathophysiology and treatment. Biol Psychiatry.

[58] Villafuerte SM, Del-Favero J, Adolfsson R, Souery D, Massat I, Mendlewicz J, Van Broeckhoven C, Claes S (2002). Gene-based SNP genetic association study of the corticotropin-releasing hormone receptor-2 (CRHR2) in major depression. Am J Med Genet.

[59] Claes S (2009). Glucocorticoid receptor polymorphisms in major depression. Ann N Y Acad Sci.

[60] Ellsworth KA, Moon I, Eckloff BW, Fridley BL, Jenkins GD, Batzler A, Biernacka JM, Abo R, Brisbin A, Ji Y, Hebbring S, Wieben ED, Mrazek DA, Weinshilboum RM, Wang L (2013). FKBP5 genetic variation: association with selective serotonin reuptake inhibitor treatment outcomes in major depressive disorder. Pharmacogenet Genomics.

[61] Binder EB, Owens MJ, Liu W, Deveau TC, Rush AJ, Trivedi MH, Fava M, Bradley B, Ressler KJ, Nemeroff CB (2010). Association of polymorphisms in genes regulating the corticotropin-releasing factor system with antidepressant treatment response. Arch Gen Psychiatry.

[62] Xiao Z, Liu W, Gao K, Wan Q, Yang C, Wang H, Wang X, Wang G, Liu Z (2011). Interaction between CRHR1 and BDNF genes increases the risk of recurrent major depressive disorder in Chinese population. PLoS One.

[63] Surget A, Belzung C (2008). Involvement of vasopressin in affective disorders. Eur J Pharmacol.

[64] Van Pett K, Viau V, Bittencourt JC, Chan RK, Li HY, Arias C, Prins GS, Perrin M, Vale W, Sawchenko PE (2000). Distribution of mRNAs encoding CRF receptors in brain and pituitary of rat and mouse. J Comp Neurol.

[65] Ventura-Juncá R, Herrera L (2012). Epigenetic alterations related to early-life stressful events. Acta Neuropsychiatrica.

[66] Duman RS, Monteggia LM (2006). A neurotrophic model for stress-related mood disorders. Biol Psychiatry.

[67] Dalton VS, Kolshus E, McLoughlin DM (2013). Epigenetics and depression: return of the repressed. J Affect Disord.

[68] Hennings JM, Owashi T, Binder EB, Horstmann S, Menke A, Kloiber S, Dose T, Wollweber B, Spieler D, Messer T, Lutz R, Kunzel H, Bierner T, Pollmacher T, Pfister H, Nickel T, Sonntag A, Uhr M, Ising M, Holsboer F, Lucae S (2009). Clinical characteristics and treatment outcome in a representative sample of depressed inpatients - findings from the Munich Antidepressant Response Signature (MARS) project. J Psychiatr Res.

